# Butyrate Supplementation Exacerbates Myocardial and Immune Cell Mitochondrial Dysfunction in a Rat Model of Faecal Peritonitis

**DOI:** 10.3390/life12122034

**Published:** 2022-12-06

**Authors:** Vera B. M. Peters, Nishkantha Arulkumaran, Miranda J. Melis, Charlotte Gaupp, Thierry Roger, Manu Shankar-Hari, Mervyn Singer

**Affiliations:** 1 Bloomsbury Institute of Intensive Care Medicine, Division of Medicine, University College London, London WC1E 6BT, UK; 2 Infectious Diseases Service, Department of Medicine, Lausanne University Hospital and University of Lausanne, CH-1066 Lausanne, Switzerland; 3 Centre for Inflammation Research, Institute for Regeneration and Repair, The University of Edinburgh, Edinburgh EH16 4TJ, UK

**Keywords:** sepsis, butyrate, nutrition, fatty acids, immunity, mitochondria

## Abstract

**Simple Summary:**

Sepsis is a major clinical problem with high incidence and mortality. While nutrition is routinely provided to critically ill patients, this essentially consists of a one-size-fits-all balanced protein-carbohydrate-fat diet given with little regard to the changing immunological, bioenergetic and metabolic status in sepsis. Besides being an important source of calories, nutrition also has a pharmacological impact. Fatty acids, such as butyrate, impact upon immune function, mitochondrial function and metabolism in different ways, and their individualised use may contribute to a personalised medicine approach depending on the patient’s immune and bioenergetic status. In this research, we combined immunomodulatory effects of butyrate with metabolism. We showed anti-inflammatory effects of butyrate in our ex vivo study on PBMCs isolated from healthy human volunteers. We took this forward in a clinically relevant faecal peritonitis rat model of sepsis with butyrate infusion, but rather demonstrated possible cardiometabolic stress with no impact on immune function.

**Abstract:**

Mitochondrial dysfunction and immune cell dysfunction are commonplace in sepsis and are associated with increased mortality risk. The short chain fatty acid, butyrate, is known to have anti-inflammatory effects and promote mitochondrial biogenesis. We therefore explored the immunometabolic effects of butyrate in an animal model of sepsis. Isolated healthy human volunteer peripheral mononuclear cells were stimulated with LPS in the presence of absence of butyrate, and released cytokines measured. Male Wistar rats housed in metabolic cages received either intravenous butyrate infusion or placebo commencing 6 h following faecal peritonitis induction. At 24 h, splenocytes were isolated for high-resolution respirometry, and measurement of mitochondrial membrane potential (MMP), reactive oxygen species (mtROS), and intracellular cytokines (TNF alpha, IL-10) using flow cytometry. Isolated splenocytes from septic and septic butyrate treated rats were stimulated with LPS for 18 h and the effects of butyrate on cytokine release assessed. Ex vivo, butyrate (1.8 mM) reduced LPS-induced TNF alpha (*p* = 0.019) and IL-10 (*p* = 0.001) release by human PBMCs. In septic animals butyrate infusion reduced the respiratory exchange ratio (*p* < 0.001), consistent with increased fat metabolism. This was associated with a reduction in cardiac output (*p* = 0.001), and increased lactate (*p* = 0.031) compared to placebo-treated septic animals (*p* < 0.05). Butyrate treatment was associated with a reduction in splenocyte basal respiration (*p* = 0.077), proton leak (*p* = 0.022), and non-mitochondrial respiration (*p* = 0.055), and an increase in MMP (*p* = 0.007) and mtROS (*p* = 0.027) compared to untreated septic animals. Splenocyte intracellular cytokines were unaffected by butyrate, although LPS-induced IL-10 release was impaired (*p* = 0.039). In summary, butyrate supplementation exacerbates myocardial and immune cell mitochondrial dysfunction in a rat model of faecal peritonitis.

## 1. Introduction

Sepsis defined as life-threatening organ dysfunction caused by a dysregulated host response to infection [1], is a major global health problem [2]. The immunological response to sepsis is characterised by excessive pro- and anti-inflammatory responses, the balance of which fluctuates over the course of the illness [3]. An overall immunosuppressive state often dominates following an early pro-inflammatory phase, with both depletion and loss of functionality of both innate and adaptive immune cells increasing the risk of secondary infection [4].

Different metabolic phases in the course of sepsis and recovery are also described [5,6,7,8]. An early hypermetabolic response, with increased total body oxygen consumption, is followed by a hypometabolic phase with downregulation of many cellular metabolic activities and mitochondrial functionality [9,10]. Recovery coincides with a hypermetabolic phase with increases in oxygen and substrate utilisation to drive tissue repair and restoration of organ function [11,12]. Sepsis is also known to alter mitochondrial functionality of immune cells [13,14,15].

Butyrate is a short-chain fatty acid (SCFA) that regulates immune responses [16,17,18,19]. SCFAs have predominant anti-inflammatory effects and are involved in chemotaxis, apoptosis, proliferation, differentiation and gene expression [20]. Butyrate stimulates mitochondrial respiration and fatty acid oxidation (FAO) in several in vitro and murine studies [21,22,23], though others have shown a depressant effect on ATP synthesis [24]. We therefore examined the impact of supplemental butyrate on mitochondrial and immune function, and cardiovascular effects, in in vivo, ex vivo and in vitro models of sepsis.

## 2. Materials and Methods

### 2.1. Study Design

To investigate the effect of butyrate on immune and mitochondrial function in sepsis, corresponding in vivo and ex vivo experiments were performed:

(i) peripheral blood mononuclear cells (PBMCs) isolated from healthy volunteers were stimulated overnight with lipopolysaccharide (LPS) in the presence and absence of butyrate (Sigma-Aldrich, St. Louis, MO, USA) (1.8 mM) for 18 h. TNF alpha and IL-10 release were measured by ELISA.

(ii) butyrate (or an isocaloric placebo) infusion was commenced 6 h after induction of faecal peritonitis in an instrumented rat model receiving background fluid resuscitation. Whole body oxygen consumption (VO_2_), carbon dioxide production, and the respiratory exchange ratio (RER, ratio of CO_2_ produced to O_2_ consumed) were monitored continuously by calorimetry. At 24 h, arterial blood gas analysis and cardiac echocardiography were performed under anaesthesia followed by sacrifice of the animals to enable splenic removal. After isolation of splenocytes. measurements were made of intracellular cytokine (TNF alpha, IL-10) production (using flow cytometry), mitochondrial reactive oxygen species (mtROS) and mitochondrial membrane potential (MMP, using flow cytometry), and mitochondrial respiration (high-resolution respirometry).

(iii) Isolated splenocytes from healthy rats were incubated for 18 h in the presence of lipopolysaccharide (LPS) with TNF alpha and IL-10 and release quantified by ELISA.

### 2.2. Methodologies

More detail can be found in the Appendix A.

### 2.3. Ex Vivo Human Peripheral Blood Mononuclear Cell Isolation and Incubation

Ethics was obtained from University College London Research Ethics Committee (REC ref 19181/001). In brief, PBMCs isolated from healthy subjects were cultured for 18 h in RPMI cell culture medium (Gibco) with LPS with or without butyrate. PBMCs (300,000 cells/mL) were incubated in triplicate overnight either alone or with (i) LPS (100 ng/mL, Invivogen) in the presence or absence of butyrate (1.8 mM). This concentration was based on the three-fold elevation in plasma butyrate concentration measured in human sepsis [25]. At the end of the incubation period, plates were centrifuged and supernatants collected to quantify TNF alpha and IL-10 release by ELISA, and cellular viability was assessed using the MTT assay (Thermo Fisher Scientific, Waltham, MA, USA).

### 2.4. In Vivo Rat Model of Sepsis

**Figure 1:**
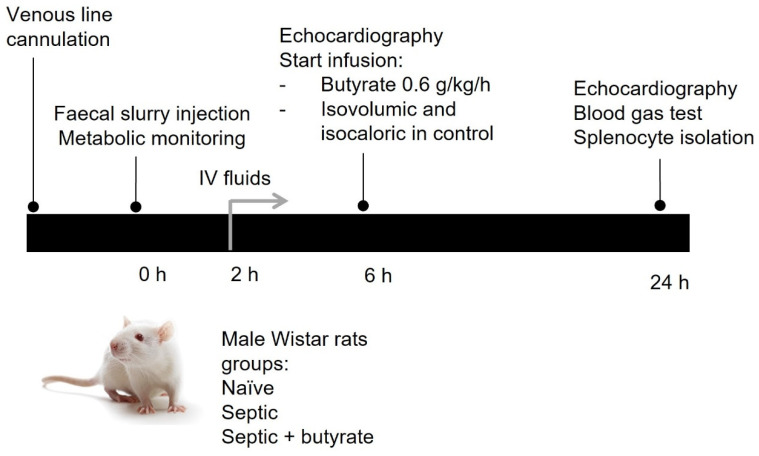
Schematic overview of rat model of sepsis.

A well-established fluid-resuscitated rat model of faecal peritonitis was used for this study [26,27]. All experiments were performed in accordance with relevant guidelines and regulations under a Home Office Project License (PPL 70/7029) and local UCL Ethics Committee approval. An overview is presented in Figure 1.

In brief, male Wistar rats weighing between 300–450 g were acclimatised overnight in separate metabolic cages allowing continuous monitoring of oxygen (O_2_) consumption and carbon dioxide (CO_2_) production. Under brief isoflurane anaesthesia, animals underwent tunnelled right internal jugular cannulation and induction of sepsis using an intraperitoneal (IP) injection of 6 μg/kg of a faecal slurry preparation. A healthy control group of animals received no injection. Animals were returned to their metabolic cages. At two hours, fluid administration was commenced using a 1:1 ratio of 5% glucose/Hartmann’s solution infused at a rate of 10 mL/kg/h.

At 6 h, echocardiography (Vivid-I, GE Healthcare) was performed under isoflurane anaesthesia to measure baseline heart rate, stroke volume and cardiac output. Following this, the septic animals received a fluid bolus of 10 mL/kg administered over five minutes followed by an infusion of sodium butyrate (0.6 g/kg/h, Sigma-Aldrich, St. Louis, MO, USA) given at a rate of 10 mL/kg/h for 18 h. The concentration selected was based on a pharmacokinetic study in mice and rats suggesting it would generate a concentration of 1.8 mM butyrate in peripheral blood [28]. Septic control animals received an isovolumic and isocaloric glucose/Hartmann’s solution.

At 24 h, echocardiography was performed under isoflurane anaesthesia followed by sacrifice by cardiac puncture with sampling of blood for measurement of lactate, glucose, and pH. The spleen was removed for ex vivo analyses.

### 2.5. Rat Splenocyte Isolation and Culture

Spleens were removed from healthy and septic animals under terminal anaesthesia. Spleens were passed through a 100 µm cell strainer and collected in RPMI cell culture medium. The cell suspension was centrifuged and red blood cells were lysed for 10 min in 1× NH_4_Cl lysing solution. Splenocytes were washed with PBS, resuspended in RPMI cell culture medium and then underwent high resolution respirometry and flow cytometry to assess mitochondrial function and intracellular cytokine production.

In separate studies, the isolated splenocytes from healthy and septic animals described above were suspended in RPMI cell culture medium, seeded into 96-well plates at a density of 300,000 cells per ml, and stimulated overnight with 100 ng/mL lipopolysaccharide (LPS) (Invivogen, San Diego, CA, USA). Viability was measured by trypan blue staining. Supernatants were stored for subsequent cytokine analyses (TNF alpha, IL-10) by ELISA.

### 2.6. High-Resolution Respirometry

Isolated rat splenocytes underwent high-resolution respirometry (Seahorse XFe96 Analyzer, Agilent Technologies, Santa Clara, CA, USA) following the manufacturer’s instructions [29]. Oxygen consumption rate (OCR) was measured at baseline (for total mitochondrial and non-mitochondrial respiration), following addition of the uncoupler, FCCP (for maximal respiration), and then addition of the electron transport chain inhibitors, rotenone and antimycin A (to block mitochondrial respiration). Three measures were made at each timepoint and averaged. Data were normalised to number of viable cells assessed by Hoechst dye (Thermo Fisher Scientific, Waltham, MA, USA) using the ImageXpress Micro XL system with MetaXpress software version 5.3.0.5 (Molecular Devices, San Jose, CA, USA). This protocol enabled calculation of basal and maximal mitochondrial respiration, non-mitochondrial respiration and proton leak.

### 2.7. Flow Cytometry

Isolated rat splenocytes were resuspended in HBSS and stained with fixable violet live/dead (Thermo Fisher Scientific, Waltham, MA, USA). MitoSOX red (2.5 μM, Thermo Fisher Scientific, Waltham, MA, USA) or tetramethylrhodamine methyl ester (TMRM; 25 nm, Thermo Fisher Scientific, Waltham, MA, USA) were added to measure mitochondrial ROS production and mitochondrial membrane potential, respectively, after 20 min’ incubation at 37 °C in 5% CO_2_.

For intracellular cytokine quantification, cells were stained with fixable violet live/dead and fixed and permeabilised using fix/permeabilize solution (BD Biosciences, Franklin Lakes, NJ, USA) for 20 min at room temperature in the dark. Cells were washed with permeabilization/wash buffer (BD Biosciences, Franklin Lakes, NJ, USA) and stained with antibodies directed against TNF alpha (Thermo Fisher Scientific, Waltham, MA, USA) and IL-10 (BD Biosciences, Franklin Lakes, NJ, USA) for another 20 min at room temperature in the dark prior to flow cytometry.

**Figure A1:**
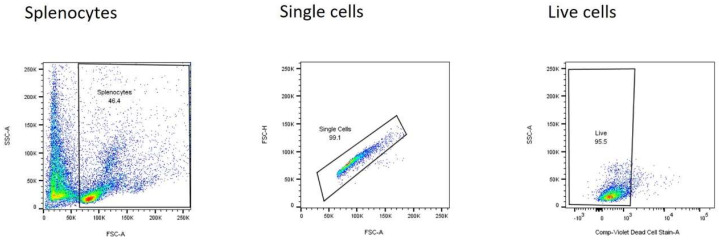
Gating strategy for in freshly isolated rat splenocytes. Blue: low event (cell) frequency, Red: high event (cell) frequency.

Flow cytometry data was analysed using FlowJo (version 10.7.1, BD Biosciences, Franklin Lakes, NJ, USA). After the exclusion of doublets and debris, live cells were analysed for mitochondrial markers and intracellular cytokine production and quantified by geometric means of the mean fluorescence intensity (MFI; arbitrary units) (Appendix A Figure A1).

### 2.8. Measurement of Released Cytokines

TNF alpha and IL-10 concentrations in cell culture supernatants were determined by ELISA according to the manufacturers’ protocols (BD Biosciences, Franklin Lakes, NJ, USA and R&D Systems, Minneapolis, MN, USA respectively).

### 2.9. Statistical Analysis

GraphPad Prism version 8.4.0 (GraphPad Software, San Diego, CA, USA) was used for statistical analyses and graphs. Non-parametric tests were performed. Data are presented as scatter plots with the median (horizontal bar). Wilcoxon tests were used for comparison between two groups. Kruskal–Wallis and Friedman tests were used to compare variables between more than two groups for unpaired and paired data, respectively. A *p*-value < 0.05 was taken as statistically significant.

## 3. Results

### 3.1. Butyrate Impaired Cytokine Release in Human Peripheral Blood Mononuclear Cell Studies

**Figure 2:**
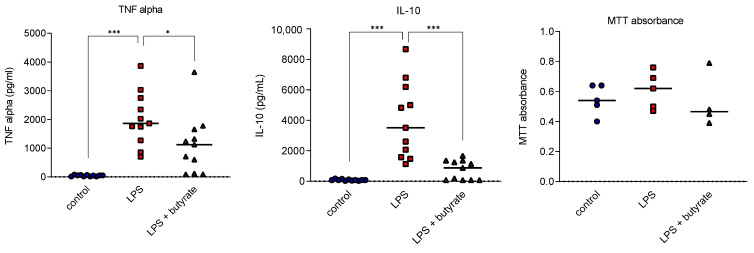
Impact of butyrate on cytokine release and MTT absorbance by human PBMCs after overnight exposure to LPS. Each symbol represents one healthy human donor (blue circle: control; red square: LPS; purple triangle: LPS + butyrate). Bar at median. * *p* < 0.05, *** *p* < 0.001.

Human PBMCs incubated with cell culture medium only produced low levels of cytokines and addition of LPS increased both TNF alpha (*p* = 0.001) and IL-10 (*p* = 0.001) release. Butyrate significantly decreased both TNF alpha (*p* = 0.019) and IL-10 (*p* = 0.001) release induced by LPS. PBMC viability was not altered by either LPS stimulation or butyrate treatment (Figure 2).

### 3.2. In Vivo Rat Model of Sepsis

**Figure 3:**
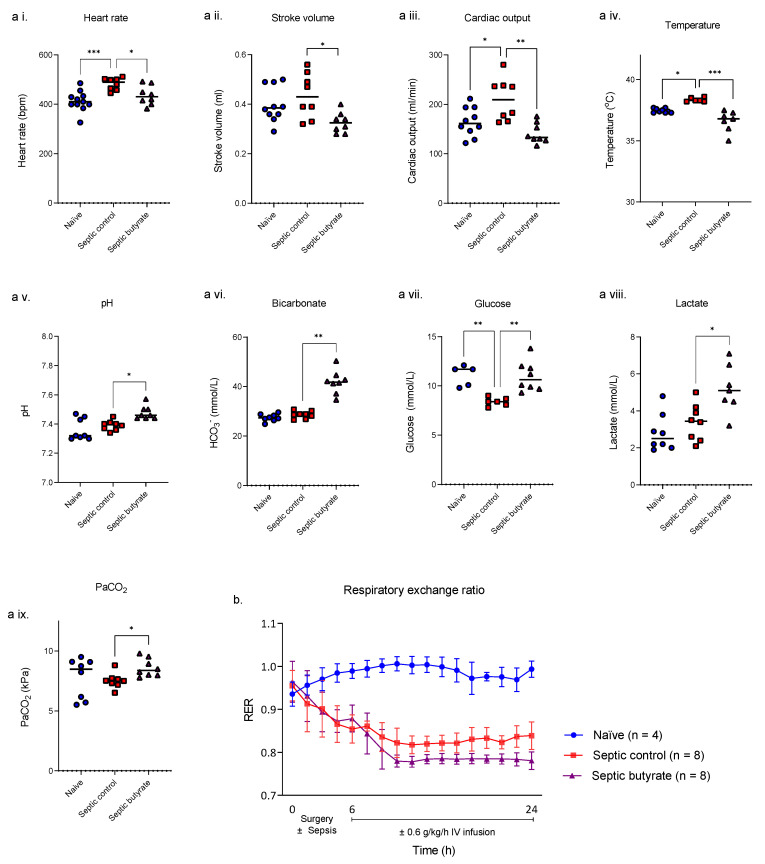
Echocardiographic, blood gas and respiratory exchange ratio (RER) for naïve, septic control and septic butyrate infused rats. Rats were either unoperated (naïve, blue circles) or injected with 6 μg/kg of faecal slurry preparation (septic) with or without infusion of 0.6 g/kg/h sodium butyrate started 6 h after the onset of faecal slurry infection (septic control, red squares; septic butyrate-treated, purple triangles). Cardiac and metabolic parameters and temperature were collected 24 h after the infectious challenge (**a**, heart rate (**a i.**); stroke volume (**a ii.**); cardiac output (**a iii.**); temperature (**a iv.**); pH (**a v.**); bicarbonate (**a vi.**); glucose (**a vii.**); lactate (**a viii.**) and PaCO_2_ (**a ix.**)). Each symbol represents one rat, and the horizontal bar the median. * *p* < 0.05, ** *p* < 0.01, *** *p* < 0.001. Continuous RER measurements started after surgery (mean ± SD) (**b**). Blue circles: naïve; red squares: septic controls; purple triangles: septic butyrate-treated. *p* < 0.001 naïve vs. sepsis from 6–24 h. *p* = 0.0005 septic control vs. septic butyrate at 24 h.

While butyrate at a concentration of 1.8 mM showed anti-inflammatory effects in our human PBMC model, we took this dose forward in our rat model of faecal peritonitis. Survival in both placebo- and butyrate-treated animals at 24 h was 100%. Compared to naïve animals, sepsis increased heart rate (*p* < 0.001), temperature (*p* = 0.015), decreased glucose (*p* = 0.003) (Figure 3a) and decreased the respiratory exchange ratio (RER) from 0.99 to 0.84 (*p* < 0.001) (Figure 3b).

Compared with untreated septic animals, at 24 h septic rats receiving butyrate showed falls in stroke volume (*p* = 0.010) and cardiac output (*p* = 0.001) with a metabolic alkalosis indicated by increases in pH (*p* = 0.010) and bicarbonate concentration (*p* = 0.003). Core temperature was lower (*p* < 0.001) while lactate levels (*p* = 0.031) were significantly higher (Figure 3a). Butyrate infusion also increased fatty acid metabolism in septic animals as indicated by a further fall in respiratory exchange ratio from 0.84 to 0.78 (*p* < 0.001) at 24 h (Figure 3b).

### 3.3. Butyrate Infusion Increased Mitochondrial Stress in Isolated Splenocytes

**Figure 4:**
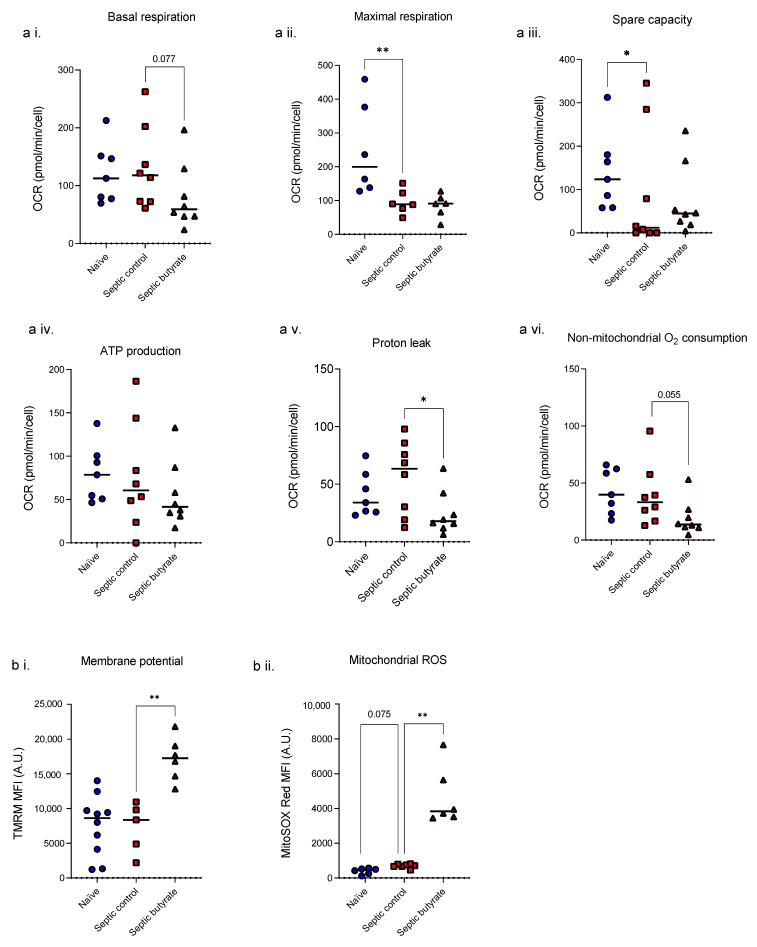
Mitochondrial respiratory variables (**a**; basal respiration (**a i.**); maximal respiration (**a ii.**); spare capacity (**a iii.**); ATP production (**a iv.**); proton leak (**a v.**); non-mitochondrial O_2_ consumption (**a vi.**)), membrane potential (**b i.**) and ROS production (**b ii.**) of rat splenocytes isolated from healthy rats or after 24 h of sepsis. Each symbol represents one rat. Blue circles: naïve; red squares: septic controls; purple triangles: septic butyrate-treated. Bar at median. * *p* < 0.05, ** *p* < 0.01.

Splenocytes isolated from animals at 24 h following induction of sepsis showed falls in maximal respiration (*p* = 0.008) and spare capacity (*p* = 0.048) compared to non-operated healthy controls (Figure 4a). Butyrate treatment given to the septic animals decreased basal respiration (*p* = 0.077), proton leak (*p* = 0.022) and non-mitochondrial oxygen consumption (*p* = 0.055). Sepsis increased splenocyte mtROS (*p* = 0.075) but had no effect on mitochondrial membrane potential; addition of butyrate however increased both membrane potential (*p* = 0.007) and mtROS (*p* = 0.027) (Figure 4b).

### 3.4. Butyrate Infusion Did Not Impact upon Sepsis-Induced Increased Intracellular Cytokine but Reduced LPS- Induced IL-10 Release

**Figure 5:**
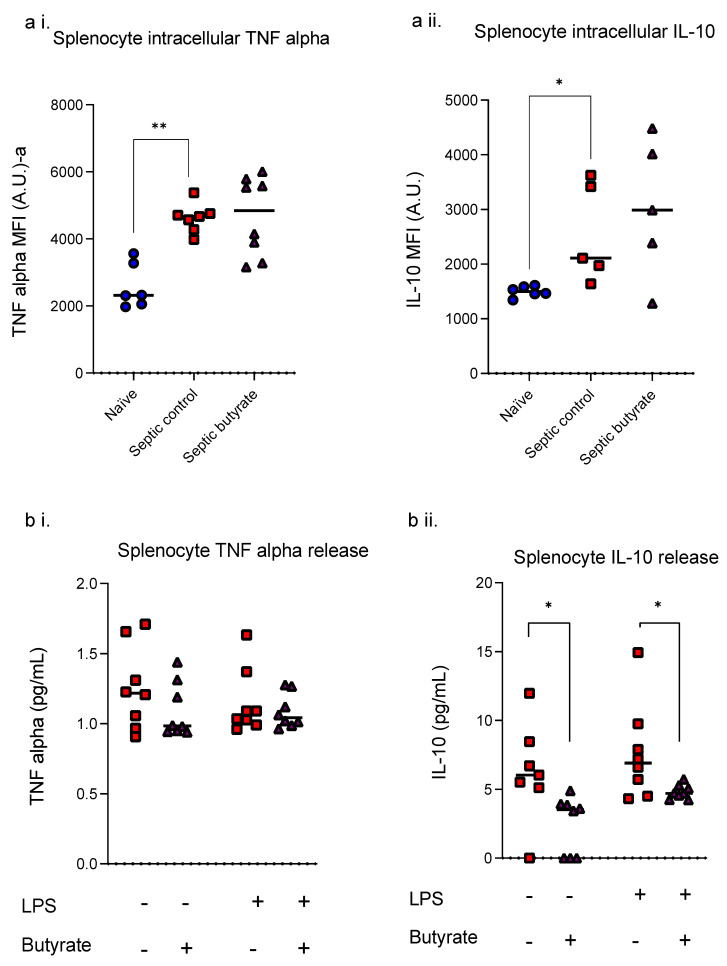
Intracellular cytokine levels (**a**; TNF alpha (**a i.**); IL-10 (**a ii.**)) and released cytokines after LPS stimulation (**b**, TNF alpha (**b i.**); IL-10 (**b ii.**)) of splenocytes isolated from healthy control rats (blue circles), septic control rats (red squares) and butyrate-treated septic rats (purple triangles) at 24 h. Each dot represents one rat. Bar at median. * *p* < 0.05, ** *p* < 0.01.

Intracellular TNF alpha was increased (*p* = 0.004) in splenocytes isolated from septic animals at 24 h compared to healthy controls but no change was seen in IL-10 levels (Figure 5). Butyrate treatment had no impact on intracellular TNF alpha and IL-10 levels.

Cytokine release by splenocytes was affected by butyrate with a reduction in released IL-10 in unstimulated (*p* = 0.031) and LPS-stimulated (*p* = 0.039) cells. TNF alpha release was unaffected by butyrate treatment. LPS stimulation on its own failed to induce TNF alpha and IL-10 release by isolated splenocytes from septic animals.

## 4. Discussion

Sepsis induces derangements in metabolic, immune and mitochondrial function. Here, we showed ex vivo, butyrate reduced LPS-induced cytokine (TNF alpha and IL-10) release by human PBMCs. We therefore took this forward in our rat model of faecal peritonitis, were we showed that intravenous butyrate infusion reduced the respiratory exchange ratio (*p* < 0.001). This was associated with a reduction in cardiac output, and increased lactate compared to placebo-treated septic animals (*p* < 0.05). Butyrate treatment did not affect mitochondrial respiration nor intracellular cytokine production in splenocytes after 24 h of sepsis, although both MMP and mtROS increased. Butyrate lowered splenocyte IL-10 release following LPS stimulation ex vivo. However, LPS stimulation on its own failed to induce TNF alpha and IL-10 release by isolated splenocytes from septic animals, which may indicate an immune exhaustion phenotype that is not restored by butyrate.

Derangements in metabolic, immune and mitochondrial function were observed in our established rat model of faecal peritonitis. Intravenous butyrate infusion however failed to show any obvious clinical benefit at a whole-body level in our established rat model of faecal peritonitis. Butyrate infusion was associated with decreased cardiovascular function and higher lactate levels. While this may indicate a deleterious effect of this SCFA, conversely a counter-argument may be made towards normalisation of cardiovascular parameters with butyrate, and the rise in lactate explained by substrate reprioritisation away from glucose and by the induced alkalosis (see below). Though mitochondrial respiration and total body oxygen consumption were suppressed at 24 h, there was an increase in splenocyte mitochondrial reactive oxygen species levels. Others have reported that SCFA supplementation (including butyrate) reduced inflammation and increased survival in septic rats [30], but we were unable to reproduce this effect. Many of these prior studies however differed in setup or did not deliver a realistic in vivo septic insult but instead administered a single injection of LPS [31]. Butyrate was also given by intermittent injection [30] rather than by continuous infusion, or as pre-treatment, either in the feeding regimen [32] or intraperitoneally [33] which does not follow a clinical setting where patients first get ill and then receive treatment. Fluid resuscitation was also highly variable and continuous infusions were not administered. In clinical practice, treatment (e.g., fluid resuscitation, immunomodulatory therapy) would not commence until the patient presents with established sepsis. In this context, our model better reflects the clinical scenario. In our model we attempted to mimic a more realistic clinical scenario, with butyrate administration commenced at 6 h after the induction of sepsis when animals were manifesting clinical features, plus maintenance of an adequate volaemic status.

In our septic model there was a significant reduction in RER, indicating a shift towards lipid metabolism. This was exacerbated by infusion of butyrate. In a study performed in septic or trauma patients, a large glucose load did not impact upon fat oxidation but did increase conversion of glucose to glycogen [34]. FA uptake and blood levels were not measured. The higher blood glucose level seen in our study in butyrate-treated septic rats suggests increased utilization of fat and, perhaps, more insulin resistance. Similar findings were reported in a dog sepsis model [35]; butyrate was infused in the form of the ketone body, β-hydroxybutyrate but this failed to suppress glucose production or lower plasma free FAs.

The rise in lactate seen with butyrate treatment may reflect worse tissue perfusion and/or mitochondrial impairment of oxidative phosphorylation. Depressed cardiac function is a major complication of sepsis [36] yet cardiac output was lowered further with butyrate treatment. These results differ from those from obtained in a murine endotoxin model [37]. A butyrate diet, given as pre-treatment, attenuated septic myocardial depression, and this was associated with an anti-inflammatory effect and a reduction in oxidative stress. An additional cause of hyperlactataemia is the significant metabolic alkalosis induced by butyrate in the septic rats. MacLeod and Hoover observed a century ago that alkalosis, either metabolic or respiratory, increases aerobic production of lactate [38]. The alkalosis-induced rise in lactate production is linked in part to the sensitivity of phosphofructokinase (PFK). PFK is involved in the sequential conversion of glucose to lactate (Embden-Meyerhof pathway) and enhances glycolysis [39]. Alkalosis may impair mitochondrial oxidation and the activity of the Krebs’ cycle [40].

We observed that butyrate increased both mitochondrial membrane potential and mitochondrial reactive oxygen species within splenocytes isolated from the septic rats. Unexpectedly, however, there was an overall depressant effect on bioenergetics. Butyrate is generally associated with increased mitochondrial respiration [21] though decreased ATP turnover has been reported [24]. By contrast, butyrate treatment was associated with lower splenocyte proton leak compared to untreated septic rats, suggesting that this SCFA decreased mitochondrial uncoupling. Uncoupling may elevate core temperature which was blocked by butyrate treatment. Uncoupling will also reduce mitochondrial ROS production through decreasing mitochondrial membrane potential. The underlying redox status may be relevant in determining whether butyrate treatment is protective or potentially damaging; opposing effects may be seen in health or stressful conditions such as sepsis. Of note, FAs reduced ROS generation due to their weak inhibition of electron flow at complexes I and III [41] and their protonophore action on the inner mitochondrial membrane [42]. The latter effect increases as FA chain length increases. SCFAs such as butyrate, which has a chain length of 4 carbon atoms, will thus have less protonophore activity. Low physiological levels of butyrate did not affect functioning of the electron transport chain nor did it have any protonophore effects on the inner mitochondrial membrane [41].

Another consideration is dose dependency, since butyrate may have variable effects on respiration depending on the dose [43]. We took forward our dose of 1.8 mM as this concentration was based on the three-fold elevation in plasma butyrate concentration measured in human sepsis [25], and was effective in our ex vivo human PBMC study. A pharmacokinetic study in mice and rats suggested our infusion of 0.6 g/kg/h butyrate would generate a concentration of 1.8 mM butyrate in peripheral blood [28]. Unfortunately, despite efforts, we were unable to measure the plasma butyrate concentrations in our septic rats. We acknowledge the possibility of toxicity which may not occur at lower doses. This may be in part related to the metabolic alkalosis generated by the butyrate infusion with median 24 h plasma bicarbonate levels rising above 40 mmol/L. While this extracellular increase may not necessarily reflect intracellular pH changes in vivo, mitochondrial function is tightly regulated by pH [44]. There is a marked pH difference in control (5.0–7.0 for Hartmann’s solution, 4.0 for 5% glucose) versus butyrate-containing (pH 7.7) fluid so there will be considerably greater buffering capacity with butyrate.

While sepsis increased intracellular TNF alpha and IL-10 production in our rat model, butyrate infusion had no effect. IL-10 release was reduced by butyrate in our ex vivo model, yet TNF alpha release remained unaltered. Although models differ, suppression of pro-inflammatory cytokine secretion by butyrate [45,46,47,48,49,50], and an increase in monocyte IL-10 secretion [48] have previously been reported. Butyrate is a potent inhibitor of nuclear factor (NF)-κB, which offers a potential explanation for the reduced IL-10 release [51,52]. Butyrate inhibits activity of histone deacetylases (HDACs) [50] and activates G protein-coupled receptors GPRs [53,54], thereby promoting Treg development and function, and shaping differential T cell responses [55], yet we did not study underlying mechanisms.

While LPS stimulation in the absence of butyrate did not increase cytokine release by isolated splenocytes from septic rats, this may indicate immune cell exhaustion (i.e., sepsis induced immunosuppression). We have not however explored this in our study. Boomer et al. reported a profound depletion of T, B and dendritic cells in both murine models of sepsis [56,57] and septic patients [58,59,60,61]. Post-mortem analyses of spleen and lymph nodes taken from patients who died from sepsis showed a significant loss of CD4+ and CD8+ T cells [59]. Additionally, T cells showed increased expression of the immune checkpoints, programmed cell death 1 (PD-1) and cytotoxic T-lymphocyte-associate antigen-4 (CTLA-4) [59], possibly accounting for the increased lymphocyte death and cellular unresponsiveness to inflammatory stimuli [62]. Similarly, B and T lymphocyte attenuator (BTLA) and programmed death ligand (PD-L1) are upregulated in B cells, and monocytes [58,59]. Furthermore, expression of CD28 is decreased in T cells isolated from septic patients compared to healthy controls [59]. Without co-stimulation through CD28/B7 when the T cell receptor engages antigen, T cells become functionally unresponsive [63]. Our data are consistent with immune cell exhaustion, although we have not studied this. Treatment with butyrate did not affect these changes.

Future work should analyse the impact of potential confounders such as blood (extracellular) pH. Other investigations could include mechanistic studies such as expression or activity of NF-κB, HDACs, GPRs or uncoupling proteins (UCPs). UCP2 is expressed on immune cells and involved in cell activation, mitochondrial ROS production and fatty acid oxidation [64]. The effects of butyrate could be tested on peripheral blood purified monocytes or lymphocytes rather than isolated splenocytes, though recognising that cell numbers fall markedly during sepsis so adequate yields may become problematic. Comparisons between cell types may reveal important differences, and addition of immunosuppressive markers including PD-(L)1, CTLA-4 and/or HLA-DR may provide further insight. Our rat model has been long established to mimic a clinically relevant sepsis situation with impaired mitochondrial function and myocardial depression [26,27]. However, preclinical models including ours generally use young and healthy animals while sepsis predominantly affects elderly co-morbid patients with impaired immune functionality.

## 5. Conclusions

Although intravenous butyrate infusion reduced the inflammatory response in our rat model of faecal peritonitis-induced sepsis, supplemental butyrate may be associated with deleterious effects in early sepsis as butyrate administration was associated with cardio depressant effects, increased lactate levels, increased splenocyte ROS, and impaired LPS-induced IL-10 release. Hence, the therapeutic utility of butyrate is doubtful. Further studies are needed to determine whether the butyrate regimen given to this model was optimal.

## Appendix A. Detailed Methods

### A.1. Butyrate Stock Preparation

Butyrate stock solution (100 mM) was prepared by dissolving butyrate powder (Sigma-Aldrich, St. Louis, MO, USA) into prewarmed distilled water. Stocks were kept at −20 °C until use for human PBMC experiments.

### A.2. Human PBMC Isolation and Culture

Venous blood from healthy volunteers was obtained by phlebotomy and diluted 1:3 with PBS. Diluted blood was layered on Ficoll-Paque (GE Healthcare, Uppsala, Sweden) and centrifuged for 30 min at 400× *g* without brake at room temperature. The PBMC layer was transferred into a clean tube and washed twice with PBS, after which the cells were counted and used in experiments. Freshly isolated PBMCs from healthy donors were cultured in RPMI 1640 GlutaMAX medium supplemented with 10% fetal bovine serum and 1% penicillin/streptomycin (all Thermo Fisher Scientific Waltham, MA, USA) in a round bottom 96-wells plate at 37 °C in 5% CO_2_ at a density of 300,000 cells/mL.

### A.3. MTT Assay

Viability of human PBMCs was confirmed by using the MTT assay. MTT solution was prepared by dissolving MTT dye in IMDM (Thermo Fisher Scientific Waltham, MA, USA, 1 mg MTT per ml IMDM). At the end of incubation, supernatant was collected as described before and cells then incubated for 2 h at 37 °C, 5% CO_2_ with 100 μL MTT solution per well. MTT solution was removed and 100 μL lysing solution (a mixture of isopropanol, SDS 20% and HCl 5 N, ratio 10:5:0.1) per well was added to dissolve the cells. Plates were covered and incubated overnight on a plate shaker. Absorbance was measured at 570 nm.

### A.4. In Vivo Rat Model of Sepsis

Male Wistar rats (Charles River UK) weighing between 300–450 g were acclimatised overnight in separate metabolic cages (CLAMS Oxymax, Columbus Instruments, Ohio, PA, USA), allowing continuous monitoring of oxygen (O_2_) consumption and carbon dioxide (CO_2_) production. Under 2% isoflurane anaesthesia the right jugular vein was cannulated and tunnelled to exit at the nape of the neck. Sepsis was induced by an intraperitoneal (IP) injection of 6 μg/kg of a faecal slurry preparation dissolved in 3–4 mL of 0.9% saline. Control animals received no injection. A subcutaneous injection of 0.5 mg/kg buprenorphine (Vetergesic, Reckitt Benckiser, Slough, UK) was given at the time of surgery to provide long-acting analgesia. Animals were then put back in their metabolic cages and allowed to recover. At two hours, a fluid (1:1 ratio of 5% glucose/Hartmann’s solution) infusion was started at a rate of 10 mL/kg/h. At six hours, echocardiography (Vivid-I, GE Healthcare) was performed under isoflurane anaesthesia to measure heart rate, stroke volume and cardiac output.

At this 6 h timepoint, a fluid bolus of 10 mL/kg administered over five minutes followed by an infusion of sodium butyrate (0.6 g/kg/h, Sigma-Aldrich, St. Louis, MO, USA) at a rate of 10 mL/kg/h for the following 18 h. To match the caloric intake of the butyrate- treated animals, control animals received an isovolumic and isocaloric glucose/Hartmann’s solution by increasing the glucose:Hartmann’s ratio.

At 24 h, echocardiography was performed under isoflurane anaesthesia followed by sacrifice by cardiac puncture with sampling of blood and splenic tissue. Arterial blood gas was performed to measure lactate, glucose, and pH.

### A.5. Rat Splenocyte Isolation

After sacrificing the animal by cardiac puncture, the complete spleen was isolated and put in a Petri dish filled with fridge-cold culture medium. The spleen was broken in pieces in the Petri dish and then passed through a 100-μm cell strainer to obtain a single cell suspension by crushing with a 5 mL syringe and collecting the cell suspension in 5 mL culture medium. Culture medium was added on top of the strainer to keep the tissue under medium throughout. The cell suspension was centrifuged at 4 °C, 130 RCF for 5 min. Cells were resuspended in 15 mL 1× NH_4_Cl lysing solution, incubated at room temperature for 10 min and centrifuged again to lyse and wash out erythrocytes. Splenocytes were washed twice in 15 mL PBS and re-suspended in culture medium.

### A.6. Respirometry

The Seahorse XFe96 Analyzer (Agilent Technologies, Santa Clara, CA, USA) was used according to the manufacturer’s protocol to measure the oxygen consumption rate (OCR) of isolated rat splenocytes. Splenocytes were seeded in triplicate at a density of 300,000 cells per well. After overnight incubation, plates were centrifuged and supernatant was stored at −20 °C to quantify cytokines. Cells were resuspended in Seahorse XF DMEM medium (Agilent Technologies, Santa Clara, CA, USA) supplemented with 5 mM glucose XF (Agilent Technologies), 1 mM pyruvate XF (Agilent Technologies, Santa Clara, CA, USA) and 2 mM glutamine (Sigma-Aldrich, St. Louis, MO, USA) in specialised XF96 cell culture microplates (Agilent Technologies) pre-treated with Cell Tak (VWR international, Radnor, PA, USA). Plates were centrifuged at 200× *g* for 1 min without break and then incubated for 30 min at 37 °C without CO_2_. Plates were loaded onto the Seahorse analyser. Three baseline OCR measurements were taken for each well, after which oligomycin (2 μM, Sigma-Aldrich, St. Louis, MO, USA), carbonycyanide p-(trifluoromethoxy) phenylhydrazone (FCCP, 2 μM, Cambridge Bioscience), antimycin A and rotenone (1 μM, Insight Biotechnology) were sequentially injected. Three OCR measurements were taken after each injection. Hoechst dye (Thermo Fisher Scientific Waltham, MA, USA) was added to normalise OCR measurements according to cell number using the ImageXpress Imaging System (Molecular Devices). To calculate OCRs for basal respiration, proton leak, ATP production, maximal respiration and non-mitochondrial respiration, the area under the curve (AUC) prior to the next injection was calculated.

### A.7. Flow Cytometry

Isolated rat splenocytes were stained with fixable violet live/dead (Thermo Fisher Scientific Waltham, MA, USA) to assess viability.

MitoSOX red (2.5 μM) and TMRM (25 nM, both Thermo Fisher Scientific Waltham, MA, USA) were added 20 min prior to flow cytometry for measurement of mitochondrial reactive oxygen species (ROS) and mitochondrial membrane potential, respectively.

Intracellular cytokine staining was performed using the BD fixation/permeabilization kit as per manufacturer recommendations. Briefly, cells were resuspended in 100 µL/well fixation/permeabilization solution, incubated for 20 min at 4 °C before being washed and resuspended in permeabilization/wash buffer with intracellular cytokines TNF (Thermo Fisher Scientific Waltham, MA, USA) and IL-10 (BD Biosciences, Franklin Lakes, NJ, USA). Following incubation for 30 min at 4 °C, cells were washed and resuspended in staining buffer in preparation for analysis (Appendix A Table A1).

**Table A1:** Antibodies for flow cytometry.

Cell Marker	Cat. No	Clone	Laser	Filter	Fluorophore	Dilution
TNF-a	11-7423-82	TN3-19.12	Blue (488 nm)	530/30	FITC	1:50
IL-10	555088	A5-4	Yellow (561 nm)	585/15	PE	1:50
Live dead	L34963		Violet (405 nm)	450/50	violet	1:1000
MitoSOX			Yellow (561 nm)	585/15		2.5 µM
TMRM	T668		Yellow (561 nm)	585/15		25 nM

Isolated rat splenocytes were analysed using flow cytometry. Compensation controls were applied to all samples prior to analysis. Cell surface marker compensation controls used single-stained unstimulated healthy donor cells. Healthy donor cells for the Live/Dead (Thermo Fisher Scientific Waltham, MA, USA) single stain were heat-treated at 60 °C for 10 min.

Cells were initially gated using forward and side scatter and at least 5000 splenocyte gate events were acquired for each sample (LSR II flow cytometer and FACSDiva software, BD Biosciences, Franklin Lakes, NJ, USA). Flow cytometry data was analysed using FlowJo (version 10.7.1, BD Biosciences, Franklin Lakes, NJ, USA). Cell populations of interest were identified using the following Boolean gating strategy: lymphocytes or PBMCs, singlets, live cells, and cell surface markers (Appendix A Figure A1).

After the exclusion of doublets and debris, live cells were analysed for mitochondrial markers and intracellular cytokine production and quantified by geometric means of the mean fluorescence intensity (MFI; arbitrary units).
